# Intramedullary Threaded Nail Fixation of Distal Ulnar Fractures: The Surgical Technique and Case Series

**DOI:** 10.7759/cureus.61736

**Published:** 2024-06-05

**Authors:** Alexander D Jeffs, Andrew D Allen, Bradley J Lauck, Nathaniel C Adams, Reid W Draeger

**Affiliations:** 1 Department of Orthopaedics, Chapel Hill School of Medicine, University of North Carolina, Chapel Hill, USA

**Keywords:** orthopedic hand surgery, distal ulna fracture, outcomes, surgical technique, intramedullary threaded nail

## Abstract

Background:* *To describe the surgical technique of non-compressive intramedullary threaded nail (IMTN) fixation of distal ulnar neck fractures and present the clinical and radiographic outcomes of four patients treated with this novel technique.

Methods: At a single Level 1 Trauma Center, a retrospective review was conducted for patients with distal ulnar neck fractures treated with retrograde IMTN between 2022 and 2024. Exclusion criteria included inadequate follow-up. A single surgeon performed all procedures using percutaneous retrograde IMTN fixation through the central disc of the triangular fibrocartilage complex (TFCC). Patients initiated a range of motion (ROM) protocol two weeks post-operatively. Post-operative radiographic images were used to calculate the ratio of IMTN diameter to the distal ulnar medullary isthmus diameter proximal to the fracture site. Radiographic changes in displacement, angulation, and ulnar variance were calculated between the first and last follow-up radiographs. Functional outcomes including grip strength and ROM were collected.

Results: Four patients with distal ulnar neck fractures were treated with retrograde IMTN between 2022 and 2024. They were followed for a minimum of three months post-operatively. All were female with an average age of 65 years. All distal ulna fractures were associated with operatively treated intraarticular distal radius fractures. All patients were treated with 75 mm length and 4.5 mm diameter IMTNs. IMTN-to-Isthmus ratio was greater than 60% in all cases. Average radiographic displacement and angulation were unchanged at the final follow-up. The average ulnar variance increased by 1.2 mm. At the final follow-up, there were no post-operative complications. No cases demonstrated ulnar-sided wrist pain, nonunion, or required revision surgery.

Conclusions: Retrograde IMTN fixation is a novel surgical technique for the treatment of distal ulnar neck fractures. We found limited but promising post-operative radiographic and functional outcomes in our patients without reported ulnar-sided wrist pain, nonunion, or need for hardware removal.

## Introduction

Intramedullary screw fixation of upper extremity fractures is gaining popularity secondary to providing sufficient stability for early range of motion (ROM) through a minimally invasive approach that limits soft tissue disruption. This construct is traditionally implemented in the fixation of metacarpal fractures [1,2]. Limited in the current literature is the utilization of intramedullary threaded implants for the fixation of distal ulnar neck fractures [3,4].

Distal ulnar neck fractures most commonly occur with an ipsilateral distal radius fracture [5]. While most of these injuries can be managed nonoperatively, surgical fixation should be considered if the distal ulna remains unstable or displaced following fixation of the radius. Various techniques are described for the fixation of distal ulnar neck fractures, including Kirschner-wire (K-wire) fixation, tension band wiring, plate and screw constructs, and intramedullary headless screw (IMHS) fixation [3,4,6]. Plate fixation is well established in the literature as the gold standard for stability and union [7-10]. However, the distal ulna has a tenuous soft tissue envelope and rates of hardware removal related to plate prominence have been documented at 29% [11]. Additionally, the small size of the distal fracture fragment in relation to the large articular surface area of the ulnar head typically limits fixation options [11].

Recent intramedullary threaded nail (IMTN) implants have been engineered primarily for the treatment of metacarpal fractures, with multiple design characteristics that can be advantageous in the treatment of distal ulnar neck fractures [12-14]. The IMTN implants have a non-compressive design and provide larger diameters as well as longer length options. In comparison to alternative techniques, IMTNs can be inserted retrograde with minimal soft tissue dissection and no hardware prominence.

Despite technique descriptions for the use of IMHS fixation for distal ulna fractures, there remains a lack of literature analyzing the clinical outcomes or surgical technique of IMTNs [3,4]. The aim of this study is to describe the surgical technique of retrograde IMTN fixation for distal ulnar neck fractures and report the radiographic and functional outcomes for patients treated with this fixation method at a single institution. 

## Case presentation

Methods

**Figure 1:**
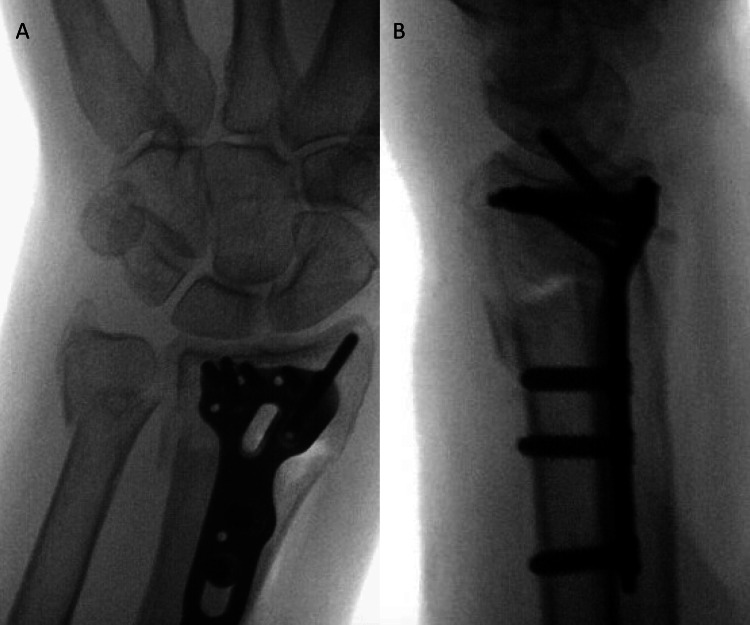
Case 3 from left to right: AP (A) and lateral (B) intraoperative fluoroscopic images demonstrating persistent instability of the ipsilateral ulnar neck fracture after volar locking plate fixation of the distal radius fracture.

We retrospectively identified all patients in the electronic medical record at a single level-one trauma center from 2022 to 2024 who were treated with a retrograde IMTN for a distal ulnar neck fracture. All cases were performed by a single surgeon. We defined a distal ulnar neck fracture as a fracture occurring within 5 cm of the dome of the ulnar head. Indications for surgery were persistent instability after fixation of the distal radius (Figure 1). Specifically, we defined this as direct palpation of instability at the fracture site and/or motion seen fluoroscopically at the fracture site, following distal radius fixation. To do this, the proximal ulna shaft is immobilized in one hand and the distal ulna segment is pinched in the other. The distal segment is then stressed in flexion/extension and pronation/supination. If the dynamic motion was palpated, directly visualized in the volar wound, or appreciated under fluoroscopy, then fixation of the ulna was indicated. Patients were excluded for age <18 years or follow-up <3 months. 

Surgical Technique

**Figure 2:**
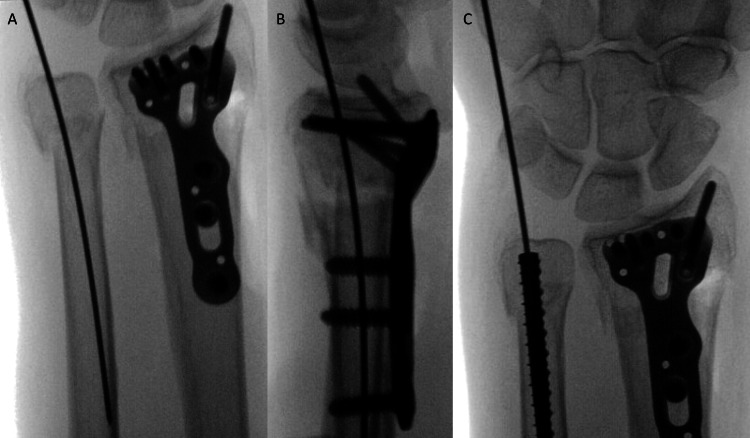
Case 3 from left to right: AP (A) and lateral (B) intraoperative fluoroscopic images demonstrating the guide wire insertion point and provisional reduction of the distal ulnar neck fracture. AP image (C) of Case 3 provisional reduction of the fracture after IMTN insertion.

The wrist is maximally radially deviated to allow for palpation of the distal ulnar styloid. After marking the ulnar styloid and identifying the soft spot volar to the ECU tendon, the entry point for the guidewire is localized with fluoroscopy to provide access to the concave portion of the ulnar head without obstruction from the carpal bones. A 1 cm incision is made at the entry site for the guidewire. After mobilizing the underlying subcutaneous tissue and ensuring the safety of the dorsal sensory branch of the ulnar nerve, a nine-inch guidewire is inserted retrograde under fluoroscopic guidance into the concave portion of the ulna head. Care is taken to ensure insertion in the region of the central disc of the triangular fibrocartilage complex (TFCC), avoiding the foveal TFCC insertion (Figure 2). This start point allows for entry through an area that is less likely to have significant sensory innervation. The starting point is confirmed on orthogonal views and the fracture is reduced. The guidewire is then passed proximal to the fracture site and retractors are placed to avoid soft tissue damage when over-drilling. Prior to insertion of the desired IMTN, the canal is reamed for the respective diameter proximal and distal to the fracture site. The IMTN is then inserted over the guidewire and advanced until the head of the implant is buried beneath the articular surface. Care is taken during IMTN insertion to avoid inadvertent advancement of the guidewire (a nine-inch or longer guidewire is helpful in avoiding this). The guidewire is then removed, and the final reduction confirmed fluoroscopic views. Care is taken to ensure the implant is sub-foveal to avoid post-operative ulnar-sided wrist pain and impaction.

Post-operative Care

Post-operatively, all patients are placed in a soft dressing and volar slab splint. They are made non-weight-bearing and discharged home the same day. Patients are immobilized until the first follow-up at 10-14 days, then they are transitioned to a custom thermoplastic volar slab splint. At this time, active ROM is initiated under the supervision of a certified occupational therapist (OT) with flexion/extension and pronation/supination as tolerated. Patients are followed at standard intervals with repeat radiographs obtained at two, six, and 12 weeks post-operatively. 

Outcome Measurements

**Figure 3:**
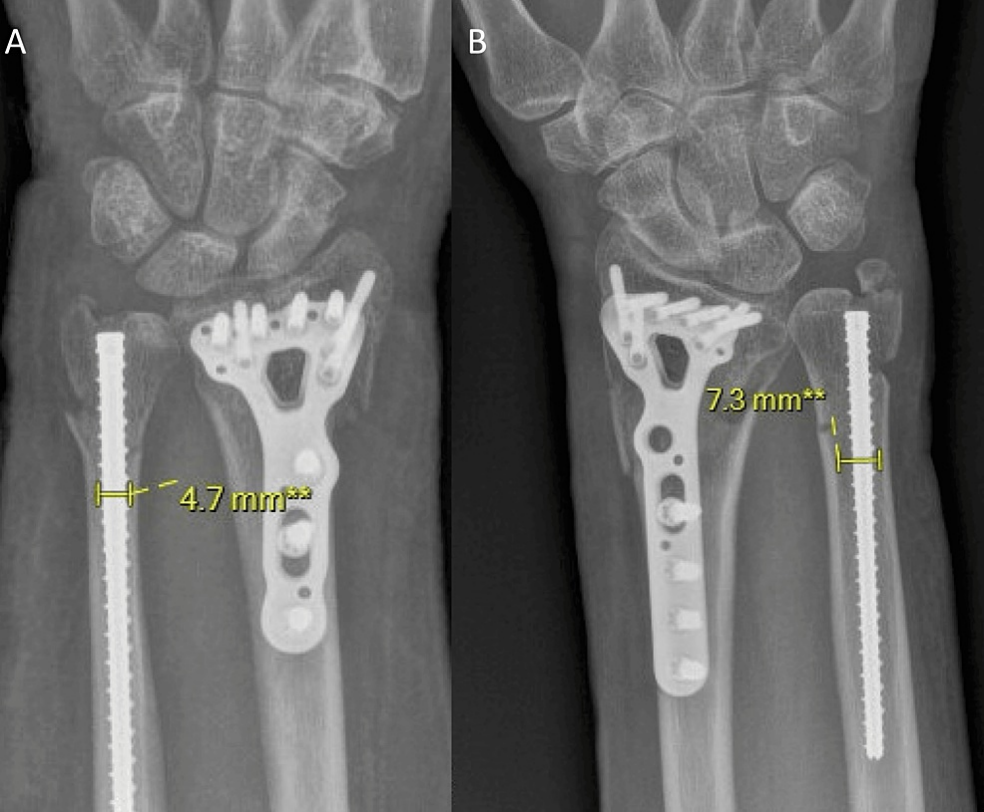
From left to right; post-operative AP radiographs of Case 2 (A) and Case 3 (B), respectively, demonstrating the distal ulna metaphyseal isthmus diameter, which was the width of the intramedullary canal measured 3 cm from the ulnar head articular surface.

The IMTN diameter to distal ulna metaphyseal isthmus diameter ratio was calculated using the AP view on the first post-operative radiograph. This ratio was defined as the screw diameter divided by the width of the intramedullary canal measured 3 cm from the ulnar head articular surface (Figure 3). This ratio was calculated to assess isthmic fill as it related to changes in ulnar variance, angulation, and displacement. Changes in radiographic fracture displacement, angulation, and ulnar variance were calculated as the difference between the first post-operative radiographs and the radiographs at the last follow-up. Displacement and angulation were calculated as the combined change in radial or ulnar translation or angulation on the AP view and volar or dorsal translation or angulation on the lateral view. The ulnar variance was calculated as the difference between the ulnar and radial length, specifically, the difference between the tangential lines of the articular surface of the ulna and the lunate fossa of the radius. Additionally, screw length past the fracture site was measured. Radiographic measurements were made with standard imaging software by a single reviewer. Functional measures were collected from OT visits including grip strength, wrist flexion and extension, and forearm pronation and supination measurements. 

Results

We identified five patients with distal ulna neck fractures who were treated with retrograde IMTN between 2022 and 2024. One patient was excluded from the analysis due to a lack of follow-up. The average time to surgery was one and a half weeks post-injury. 

Case Presentations

Case 1: A 76-year-old, right-hand dominant, female presented after a ground-level fall onto her right wrist. She sustained an impacted intraarticular distal radius fracture and a transverse distal ulna neck fracture with an associated styloid fracture. The initial displacement of the ulna fracture was 3.5 mm with 27° of angulation and neutral ulnar variance. She was indicated for distal radius fixation with a volar-locked plate and possible ulna fixation with an IMTN. 

Case 2: A 63-year-old, right-hand dominant, female presented after a ground-level fall onto her left wrist. She sustained an intraarticular distal radius fracture and an oblique distal ulna neck fracture. The initial displacement of the ulna fracture was 4.5 mm with 29° of angulation and -1.7mm of ulnar variance. She was indicated for distal radius fixation with a volar-locked plate and possible ulna fixation with an IMTN. 

Case 3: A 55-year-old, right-hand dominant, female presented after a ground-level fall onto her right wrist. She sustained an intraarticular distal radius fracture and an oblique distal ulna neck fracture with an associated styloid fracture. The initial displacement of the ulna fracture was 3.1 mm with 26° of angulation and -1.5 mm of ulnar variance. She was indicated for distal radius fixation with a volar-locked plate and possible ulna fixation with an IMTN.

Case 4: A 66-year-old, left-hand dominant, female presented after a ground-level fall onto her left wrist. She sustained transverse, comminuted distal radius and ulnar neck fractures with an associated ulnar styloid fracture. The initial displacement of the ulna fracture was 4.3 mm with 9° of angulation and +1 mm of ulnar variance. She was indicated for distal radius fixation with a volar-locked plate and possible ulna fixation with an IMTN.

Patient and Fracture Characteristics

All patients were female with an average age of 65. The average BMI was 26.6 and all patients were nonsmokers at the time of injury. Three fractures were closed injuries and one was open; all were sustained from low-energy ground-level falls. Distal ulna neck fracture morphologies included transverse (n=2) and oblique (n=2). The average initial displacement was 3.9 mm, the average initial angulation was 22.8°, and the average initial ulnar variance was -0.6 mm. All cases had a concomitant intraarticular distal radius fracture that was treated operatively with volar-locked plating at the time of distal ulna fixation. In all cases, the distal radius was fixed prior to the distal ulna. All ulna fractures were treated with a 4.5 mm diameter by 75 mm long IMTN (ExsoMed INnate Intramedullary Threaded Nail-Acumed).

Radiographic and Functional Outcomes

The average follow-up period was 22.3 weeks. All cases showed radiographic healing with sufficient bridging callus formation at a six-week follow-up. As summarized in Table 1, the average implant diameter-to-isthmus diameter ratio was 71.9%. No cases demonstrated a change in angulation or displacement between initial post-operative and final follow-up radiographs. The ulnar variance was neutral or positive post-operatively in all cases, with an average increase in the ulnar variance of +1.2 mm. Functionally, patients achieved an average final forearm pronation-supination arc of 102.5° and an average final wrist flexion-extension arc of 17.6°. 

**Table 1:** Radiographic and functional outcomes. IMTN, intramedullary threaded nail

Measurements	Case 1	Case 2	Case 3	Case 4	Average
Isthmus diameter (mm)	6.5	4.7	7.3	7.4	6.5
IMTN/isthmus ratio	0.69	0.95	0.61	0.61	0.72
IMTN length past fracture site (mm)	67.7	67	69.5	72.4	69.1
Change in ulnar variance	+1	0	+3.8	0	+1.2
Change in angulation	0	0	0	0	0
Change in displacement	0	0	0	0	0
Pronation/supination arc (°)	155	155	130	180	155
Flexion/extension arc (°)	95	120	80	115	102.5
Grip strength (% contralateral)	38.4	65.2	31.2	10.5	36.4
Final Follow-up (weeks)	51	14	12	12	22.3

## Discussion

Fractures of the upper extremity are common, with fractures of the distal radius and ulna representing the most common upper extremity fractures presenting to the emergency department [5]. Distal ulna fractures rarely occur in isolation and are usually sustained in the setting of a distal radius fracture. Most commonly, the associated ulna fracture involves only the ulnar styloid. These fractures tend to lend themselves to nonoperative management if displacement is less than 2 mm and there is no concern for distal radial ulnar joint (DRUJ) instability after fixation of the distal radius. Less common, but more difficult to manage, are fractures of the distal ulnar neck. These metaphyseal fractures more frequently necessitate surgical fixation in higher-demand patients due to persistent instability at the fracture despite the fixation of the distal radius. This is possibly secondary to the restoration of radial height, which can destabilize a distal ulnar neck fracture. Unfortunately, non-operative treatment can be associated with chronic decreased grip strength, decreased wrist/forearm ROM, and ulnar-sided wrist pain related to nonunion or malunion [8,15].

Management of an unstable distal ulnar neck fracture in a high-demand patient remains difficult due to the multi-structured articulations at the wrist involving the distal ulna, radius, carpus, and TFCC. Multiple studies have proposed nonoperative or salvage procedures as options in low-demand patients over the age of 70 [6,8]. However, appropriate management is not as clear for the young, high-demand patient. When operative treatment is pursued, the anatomy of the distal ulna and the size of the distal fragment can impede certain fixation options. Plate and screw fixation theoretically offers superior rotational control of an unstable fracture, though is limited by the degree of required soft tissue dissection, prominent hardware, and difficult implant positioning [16]. Retrograde K-wire fixation offers a minimally invasive option, but inferior stability precludes early ROM [3,6].

Unfortunately, no biomechanical studies to date are available to definitively understand the stability IMTNs provide in the distal ulna. Prior studies with similar techniques and clinical outcomes are available though. Overall, there are few reports in the literature that deal with isolated distal ulnar neck fractures. Many of the patients with distal ulna fractures in association with distal radius fractures achieve sufficient stability once the radius is reduced [11]. However, as we’ve shown, there remains a subset of patients that have persistent dynamic instability. Oh et al. described the use of a retrograde 4.5 mm diameter headless compression screw in 11 patients for the treatment of ulnar neck and head fractures [3]. All patients had high functional scores, low pain, full ROM, and no complications. Another series using a threaded intramedullary pin implant in nine patients with distal ulna fractures demonstrated good patient-reported outcome measures, union in all patients, and without the need for intramedullary implant removal [17].

We describe the use of a retrograde IMTN for the fixation of unstable distal ulnar neck fractures. Our technique, like Oh et al., affords fixation with minimal disruption of the soft tissue envelope of the distal ulna [3]. Unlike the IMHS implants used by Oh et al., IMTNs have a fully threaded, non-compressive design, which should avoid shortening during insertion [3]. This technique was effective for transverse and oblique fracture patterns. However, it should be used with caution in comminuted fractures given the potential for unplanned ulnar shortening. We observed no decrease in ulnar variance in our three cases. To our knowledge, this series is the first to report these measures in operatively treated distal ulna fractures using an intramedullary nail device [3,17]. Additional favorable features of IMTN fixation include larger diameter options than most IMHSs, which seek to improve purchase in bone endosteum. Furthermore, IMTN is designed with a smaller diameter at their leading threads, facilitating passage through a narrow isthmus. While this is less of a concern in the distal ulna with its wider isthmus and may be more relevant in the treatment of metacarpal fractures, it may contribute to its overall endosteum purchase. With longer implant options available, the IMTN may achieve superior stability compared to IMHS implants, particularly for more proximal or oblique fracture patterns. Finally, the head design, compared to IMHS implants, also allows for more distal endosteal purchase, which is important in osteoporotic metaphyseal bone.

**Figure 4:**
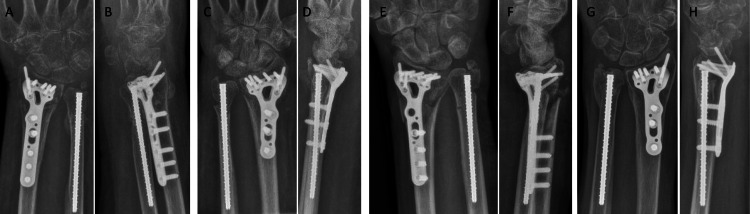
From left to right: 12-week post-operative AP (A,C,E,G) and lateral (B,D,F,H) radiographs of Cases 1 (A,B), 2 (C,D), 3 (E,F), and 4 (G,H), respectively.

Oh et al. reported an average distal ulna metaphyseal isthmus diameter of 7.4 mm [3]. Additionally, they utilized 4.5 mm diameter screws but did not report length [3]. Isthmic fill in our patients was greater than 60% in all cases and no changes in displacement or angulation were identified. Unfortunately, with our limited series size and lack of cases with displacement or malalignment, we are unable to definitively establish a threshold isthmic fill to prevent either. The literature to date offers no guidance for this and this ratio has the potential to be useful during implant selection but future studies with larger groups of patients are needed to establish it. Additionally, we did see on average 1.2 mm of ulnar positivity. It is unclear why this occurred. A potential factor could be the non-compressive design of the implant. However, there is also the likelihood of measurement error. Our clinical outcomes are limited but ulnar positivity did not seem to negatively impact ROM or impart any ulnar-sided wrist pain. Again, to better understand the significance of this, larger studies are needed. Finally, as shown in Figure 4, the 75 mm length IMTNs bypassed the fracture site proximally by 69.2 mm on average. The ability of the implants to span this distance proximal to the fracture site potentially imparted increased stability.

In our small case series, all patients showed restoration of functional wrist flexion/extension and forearm pronation/supination by three-month follow-up. This is reassuring given the concern for decreased ROM with distal ulnar neck fractures. Our lack of return of full ROM is possibly related to short-term follow-up. Additionally, no complications occurred in our patients, including nonunion, hardware failure, hardware removal, or ulnar-sided wrist pain. There is concern for the development of ulnar-sided wrist pain with this surgical technique given insertion through the TFCC near the fovea. Neimann et al. reported ulnar-sided wrist symptoms in three patients using a threaded pin implant through a retrograde ulna approach; however, we did not observe ulnar-sided wrist pain in our case series [17]. We ensured entrance into the ulnar head through the central disc where there is less sensory innervation, potentially reducing the risk of post-operative ulnar-sided wrist pain. Overall, we described the feasibility of a novel surgical technique for the fixation of metaphyseal distal ulna fractures and demonstrated satisfactory clinical outcomes in our four-patient case series.

Based on our results, this treatment is a viable option in patients greater than 65 with low-energy mechanism fractures. However, this treatment could also be viable in younger patients (<65) with high-energy fractures. The metadiaphyseal location of these fractures, proximal to the distal radial ulnar joining (DRUJ), allows for secondary bone healing, which is favorable in a high-energy extra-articular fracture pattern. This series suggests that the IMTN implant imparts the relative stability and environment necessary to achieve this. Future biomechanical studies could better establish the degree of stability imparted by the implant. Additionally, as previously suggested, entry through the central portion of the TFCC limits secondary pain generators. 

There were multiple limitations to this study. The novelty of this implant and technique limited the inclusion of patients prior to 2022 and the inclusion of patients treated by other surgeons. This case series had a small sample size and lacked direct comparison to other treatment techniques. Additionally, the design of this series was to describe a novel treatment and present outcomes with short-term follow-up from a small series of patients. Although our short-term follow-ups revealed no complications, specifically pain, studies with longer-term follow-ups at two or three years will be needed to catch any occurrences of post-traumatic DRUJ or TFCC arthritic changes. The available data was limited due to the retrospective design, with limited patient-reported outcomes data. Statistical comparisons were limited by group size, a post-hoc piori power analysis based on the work by Stock et al., showed that group sizes of at least 13 patients per group would have been necessary to achieve 80% power at an alpha error of p<0.05 [7]. Future studies should prospectively collect patients managed nonoperatively or operatively, with multiple fixation methods, to identify statistical differences in radiographic and functional outcomes among the various treatment modalities. 

## Conclusions

Retrograde IMTN fixation is a novel surgical technique for the treatment of distal ulnar neck fractures. We found limited but promising post-operative radiographic and functional outcomes in our patients without reported ulnar-sided wrist pain, nonunion, or need for hardware removal. We believe this technique to be effective in the treatment of unstable distal ulnar neck fractures after ipsilateral distal radius fixation. We recommend consideration of this technique for this difficult-to-treat fracture pattern and future studies with long-term follow-up to assess for late-onset ulnar-sided wrist pain or other complications. 
